# Quality Characteristics and Shelf-Life of Ultra-High Pressure Homogenized (UHPH) Almond Beverage

**DOI:** 10.3390/foods4020159

**Published:** 2015-05-20

**Authors:** Victoria Ferragut, Dora C. Valencia-Flores, Marianita Pérez-González, Joan Gallardo, Manuela Hernández-Herrero

**Affiliations:** Centre Especial de Recerca Planta de Tecnologia dels Aliments (CERPTA), XaRTA, TECNIO, Departament de Ciència Animal y dels Aliments, Facultat de Veterinària, Universitat Autònoma de Barcelona, Bellaterra 08193, Barcelona, Spain; E-Mails: docevaf@yahoo.com.mx (D.C.V.-F.); mar.perez.glez@gmail.com (M.P.-G.); joanjgch@gmail.com (J.G.); Manuela.Hernandez@uab.cat (M.H.-H.)

**Keywords:** ultra-high pressure homogenization (UHPH), almond beverage, shelf life, color, physical stability, volatile profile

## Abstract

The effects of ultra-high-pressure homogenization (UHPH) at 200 MPa, in combination with different inlet temperatures (55 or 75 °C) during storage at 4 °C were studied and compared with pasteurized (90 °C, 90 s) almond beverage. Microbiological analysis of the physical (particle sedimentation and color) and volatile profile of the most relevant compound in almond beverages was performed at days 1, 7, 14, and 21 of cold storage. UHPH treatment 200 at 75 °C led to higher microbiological reduction after treatment and higher stability during cold storage in almond beverages than pasteurization or UHPH 200 at 55 °C. Physical characteristics of UHPH-treated samples exhibited a high stability during storage with a stable color. Volatile compounds extracted by solid-phase microextraction were identified by gas chromatography coupled with mass spectrometry. The effect of UHPH treatment significantly (*p* < 0.05) affected the volatile profile compared with pasteurized beverages, although UHPH conditions applied produced similar effects in almond beverages. Benzaldehyde was the most abundant compound detected in all treatments. Hexanal was more abundant in UHPH-treated samples, indicating a higher lipid oxidation compared to pasteurized almond beverages.

## 1. Introduction

Almond beverage, like other vegetable beverages, is widely present in the European market as an alternative to cow’s milk, although they are not really comparable foods. The increase in consumption of these products in the last decades is related to consumers’ tendency to look for healthy foods, especially those from vegetal origin. Vegetable beverages have been promoted as healthy foods, mainly as protectors against cardiovascular diseases [1] due to the equilibrium of unsaturated and saturated fatty acids present in their composition. Moreover, they contain other beneficial bioactive compounds: antioxidants such as flavonoids, vitamin E and polyamines, fiber, and phytosterols, which reduce cholesterol absorption. In addition, a consumer sector with particular interest for vegetable beverages are those with lactose intolerance or an allergy to cow’s milk proteins.

The most conventional treatment of commercial almond beverage is UHT (ultra-high temperature, a sterilization treatment above 100 °C for several seconds) and pasteurization (heat treatments below 100 °C for several minutes). Heat treatments may cause undesirable chemical changes, which include destruction of amino acids and vitamins, browning reactions, and development of cooked flavors. On the other hand, almond beverage is a colloidal system formed by dispersed particles such as oil droplets, solid particles from raw materials, proteins, and starch granules. This complexity makes it difficult to obtain a stable product to be stored, even for a not very long time. Commonly, in conventional heat-treated vegetable milks, such as soy milk or almond beverage, particles experience sedimentation and separate from the continuous phase, causing loss of quality.

In recent years, several emergent technologies have been developed, such as ultra-high pressure homogenization (UHPH) processing. The aim of this technology is to obtain liquid foods with better quality than conventional heat treatments, which is based on the coupled effects of UHPH on hygienic preservation and colloidal stabilization.

Pressure applied in UHPH processing is in the range of 100 to 400 MPa, producing physical phenomena such as cavitation, turbulence, impact, and shear forces [2] acting on treated food. The consequences of those mechanical forces, in addition to temperature increase experienced as a consequence of heat dissipation in the high pressure valve, are the production of fine and stable colloidal dispersions [2] and microbial destruction [3,4,5]. UHPH has great potential in the food industry. In dairy products, the UHPH-treated milk was shown not only to be an alternative for direct consumption of milk [6,7], but also for derived products such as yogurt [8] and fresh cheese [9].

In a previous study [10], a prospective investigation of UHPH conditions (pressure and inlet temperature combination) on almond beverage characteristics was made. Results showed that 200 MPa combined with different inlet temperature of the product were able to produce an overall good quality almond beverage, better than that processed by conventional pasteurization. In terms of heat treated foods, pasteurization is considered a “fresh-like” product compared to other more severe heat treatments like UHT or bath sterilization. Thus, two different UHPH treatments at 200 MPa were selected with the objective of assessing their overall quality characteristics and evolution during cold storage of almond beverages compared to pasteurized.

## 2. Experimental Section

### 2.1. Preparation and Processing of Almond Beverage

Ground almond seed used in this study was kindly provided by Nectina, S.A. (Ruidoms, Tarragona, Spain). To obtain the almond beverage, 4% (w/w) almond and 0.03% (w/w) lecithin (ADM Soy Lecithin, Decatur, Sabadell, Spain) in water at 60 °C was mixed in a tank for 8 min, circulated in a colloidal mill (E. Bachiller, B.S.A. Barcelona, Spain) for 12 min, and subsequently filtered to separate the liquid phase using a 0.100 mm steel sieve. The almond beverage obtained was the base product (BP) used for subsequent treatments and as a control for comparing the effects of pasteurization or UHPH treatments. Gross composition of BP was 3.40 ± 0.11 dry matter; 2.04 ± 0.10 fat; 1.18 ± 0.10 protein; 0.12 ± 0.02 ash; and 0.08 ± 0.22 carbohydrate. Mean pH value was 7.31 ± 0.013. BP was divided into portions; one of them was pasteurized in a multi beam tubular heat exchanger (Finamat heat exchanger, Model 6500/010, Gea Finnah Gmbh, Anhaus, Germany) at 90 °C, 90 s, with previous homogenization in a double effect homogenizer (Niro Soavi, Model X68P, Parma, Italy) at 18 MPa and 4 MPa. The two other portions were UHPH treated at two different inlet temperature-pressure combinations: 200 MPa, 55 °C inlet temperature (200, 55); and 200 MPa, 75 °C inlet temperature (200, 75).

UHPH treatments were performed with a high pressure homogenizer (model FPG11300, Stansted Fluid Power Ltd., Essex, UK). This device comprises a high-pressure ceramic valve able to support 400 MPa. Inlet and outlet temperatures of almond beverage were controlled by two heat exchangers (Garvía, Barcelona, Spain) located before the machine entrance and after the second homogenization valve, respectively. Inlet temperature, temperature after the high pressure valve, and outlet temperature were monitored in all productions. Almond beverage samples were collected in sterile bottles under a laminar flow cabin adapted to the UHPH system and stored at 4 °C to analyze quality parameters during cold storage at days 1, 7, 14, and 21. A portion of samples at those different storage periods were frozen at −80 °C for further analysis of volatile compounds.

### 2.2. Microbial Analysis

Microbiological quality of almond beverage was assessed by enumerating the following microorganisms: mesophilic aerobic bacteria were counted on PCA medium (Oxoid Ltd., 96 Basingstoke, Hampshire, UK) incubated for 48 h at 30 °C. Mesophilic aerobic spore counts were assessed by heat shock at 80 °C for 10 min, quickly cooled in ice, plated on PCA medium (Oxoid), and incubated for 48 h at 30 °C. *Bacillus cereus* was enumerated in brilliance *Bacillus cereus* medium (Oxoid), supplemented with *Bacillus cereus* selective supplement (Oxoid), and incubated at 30 °C for 48 h. Enterobacteria counts were determined in violet red bile glucose agar (VRBG, Oxoid) incubated at 37 °C for 24 h.

### 2.3. Physical Stability

Almond beverage (30 g) was poured into flexible plastic tubes (32 mm diameter, 115 mm length) and centrifuged (1000× *g*, 45 min, 20 °C). After removing the liquid phase, tubes were weighted and results were expressed as % (w/w) of solid deposition at the bottom of the tubes. Samples were preserved by adding 0.04% (w/v) sodium azide (NaN_3_).

### 2.4. Color

Color determination was performed with a Hunter Lab colorimeter (MiniScan XE™, Hunter Associates Laboratory Inc., Reston, Virginia, USA), using the D65 illuminant with an angle of observation of 10°. Almond beverage samples (50 mL) were tempered to 20 °C before analysis and poured into an optically transparent glass cup that was covered with a black protector to avoid the ambient light interaction into the sample. ΔE (color difference; Equation (1)), L* (Lightness), a* (red-green component), b* (yellow-blue component), h* (hue; Equation (2)), and C* (chrome; Equation (3)) were obtained.

(1) $$\Delta \text{E}=\sqrt{\text{L}{*}^{2}+\text{a}{*}^{2}+\text{b}{*}^{2}}$$

(2) $$\text{h}*=arc\tan\frac{b*}{a*}$$

(3) $$\text{C}*=\sqrt{a{*}^{2}+b{*}^{2}}$$

### 2.5. Volatile Profiles

To determine the volatile profile of almond beverage, we utilized the method described by [11]. Volatile compounds were extracted by solid-phase microextraction and were identified by gas chromatography coupled with mass spectrometry. Separation and identification of the volatile compounds were performed using an Agilent 6890 gas chromatograph couple to a 5975 MSD mass spectrometer (Agilent Technologies, Palo Alto, CA, USA). A J&W HP-INNOWAX capillary column (60 m × 0.25 mm × 0.25 μm) with a bonded polyethylene glycol (PEG) high polarity stationary phase was used. Tentative identification of volatile compounds was achieved by comparing their mass spectra with those of mass spectra libraries [12].

### 2.6. Statistical Analysis

All results are means of three independent almond beverage productions at the pilot plant. Each analysis was made in triplicate on days 1, 7, 14, and 21. Analysis of variance (ANOVA) by the Student-Newman-Keuls method was applied for multiple comparisons of the means. Data analysis was performed with SAS9.2.3 package [13]. Differences were considered to be significant at *p <* 0.05.

## 3. Results and Discussion

### 3.1. Effect of UHPH on Microorganism and Shelf-Life Estimation

Table 1 show the growth of total aerobic meshophilic bacteria and spores, and *Bacillus cereus* during 21 days of storage at 4 °C of almond beverage samples after applying different treatments of UHPH compared with base product (BP) and pasteurized samples.

**Table 1:** Growth of total mesophilic aerobic bacteria, mesophilic aerobic spores, and *B. cereus* counts (log CFU/mL) in base product (BP), pasteurized (PA), and UHPH (200, 55 °C and 200, 75 °C) almond beverages at 4 °C over 21 days.

		Day 1	Day 7	Day 14	Day 21
Total mesophilic aerobic bacteria	BP	5.25 ± 0.65 ^a,B^	5.00 ± 0.14 ^a,B^	6.72 ± 0.23 ^a,A^	NE
	PA	3.16 ± 0.10 ^b,D^	4.07 ± 0.17 ^b,C^	4.47 ± 0.08 ^b,B^	4.80 ± 0.14 ^b,A^
	200, 55	2.92 ± 0.06 ^c,C^	3.06 ± 0.16 ^c,C^	4.90 ± 0.18 ^b,B^	5.80 ± 0.10 ^a,A^
	200, 75	2.50 ± 0.20 ^d,C^	2.22 ± 0.08 ^d,C^	2.90 ± 0.50 ^c,C^	3.40 ± 0.20 ^c,A^
Mesophilic aerobic spores	BP	3.31 ± 0.10 ^a,B^	3.23 ± 0.14 ^a,B^	4.58 ± 0.12 ^a,A^	4.80 ± 0.14 ^a,A^
	PA	2.65 ± 0.07 ^b,B^	2.47 ± 0.24 ^b,B^	2.45 ± 0.47 ^b,A^	3.23 ± 0.38 ^b,A^
	200, 55	2.28 ± 0.17 ^c,B^	3.10 ± 0.27 ^a,A^	2.80 ± 0.04 ^b,A^	2.93 ± 0.21 ^b,A^
	200, 75	1.60 ± 0.26 ^d,A^	2.17 ± 0.16 ^b,A^	2.41 ± 0.23 ^a,b,A^	2.01 ± 0.56 ^b,A^
*B. cereus*	BP	2.92 ± 0.64 ^a,C^	4.89 ± 0.51 ^a,B^	6.55 ± 0.52 ^a,A^	6.92 ± 0.27 ^a,A^
	PA	1.81 ± 0.04 ^b,A^	1.91 ± 0.34 ^b,A^	2.91 ± 0.09 ^b,A^	2.16 ± 0.04 ^b,A^
	200, 55	0.99 ± 0.08 ^c,B^	1.56 ± 0.70 ^b,A^	2.20 ± 0.70 ^b,A^	2.10 ± 0.05 ^b,A^
	200, 75	ND ^d^	ND ^c^	ND ^c^	ND ^c^

NE: Not Examined; ND: Not Detected (<0.5 CFU/mL); ^a−d^: Different superscripts in the same column are significantly different (*p* < 0.05). ^A−C^: Different superscripts in the same row are significantly different (*p* < 0.05).

All microbial groups showed a significant decrease at day 1 for almond beverage treated at 200 MPa, 55 °C and 200, 75 °C and pasteurized compared with base product (200, 75 °C > 200, 55 °C > PA > BP). Enterobacteria counts were not detected in any sample (detection limit < 0.5 CFU/mL) (data not shown), similar to what was found by other authors [5] in soy milk treated at the same conditions and in cow milk [14]. In this study coliforms were completely inactivated by both pasteurization and UHPH treatment (200 and 300 MPa). However, in milk, coliforms were not reduced after treatment of raw cow milk at 200 MPa and 55 °C [5].

The mean value of mesophilic bacteria counts observed in BP was 5.25 log CFU/mL. Among them, the predominant groups were probably spores and *B. cereus* (3.31 log and 2.92 log CFU/mL, respectively), which is in accordance with previous surveys reported in literature [5,10] in soy milk and almond beverage. Microbial counts of samples UHPH-treated 200, 55 °C were significantly different from those pasteurized samples although UHPH treatment 200, 75 °C was the most effective against all the bacterial groups. A total reduction of *B. cereus* was obtained and significant differences (*p* < 0.05) were observed among these samples and UHPH 200, 55 °C and pasteurized samples.

After cold storage, BP samples showed an increase in mesophilic bacterial total counts (from 5.25 to 6.72 log CFU/mL at day 14) as well as spores (from 3.31 to 4.8 log CFU/mL) and *B. cereus* (from 2.92 to 6.92 log CFU/mL). Total bacterial count, spores and *B. cereus*, increased progressively in pasteurized and UHPH 200, 55 °C samples during storage at 4 °C. In general, no significant differences (*p* ≥ 0.05) were observed between counts in UHPH 200, 55 °C samples and those observed in the PA. An exception was found on day 21, when total mesophilic bacteria counts were significantly higher in UHPH 200, 55 °C samples than in pasteurized ones. The lack of difference between UHPH 200, 55 °C and PA was probably due to the recovery of healthy and sub-lethally injured vegetative cells during storage or by spore germination that remained after treatment. This recovery was not observed in UHPH 200, 75 °C. A complete microbial inactivation in soy milk at 200 MPa and inlet temperature 75 °C had been found previously [5]. Nevertheless, this treatment did not cause total inactivation in this study. This may be due to the different composition of soy milk and almond beverage and the different native microbiota.

We can conclude that increasing the inlet temperature to 75 °C is much more effective in preventing the proliferation of the spoilage microbiota of almond beverage and consequently in extending the shelf life of the product.

### 3.2. Physical Stability

This study was assessed by measuring the percentage (w/w) of solid sedimentation after centrifugation of samples. This parameter is related to the potential destabilization of dispersed particles and is the maximum value of sedimentation that could be produced in forced conditions. In parallel, the observation of spontaneous destabilization of almond beverages during storage was made.

Almond beverage is a water extract of almonds in the form of an oil-in-water emulsion. The most relevant functional property of proteins in this system is to cover oil droplets of the lipid fraction for maintaining a good dispersability of those in the continuous phase during storage. In spite of this, creaming of oil droplets and sedimentation of solid particles are the primary mode of destabilization of vegetable beverages. Both phenomena are dependent to a great extent on particle size distribution [15,16,17].

Particles in vegetable beverages include not only fat globules but also small particles, such as protein bodies and aggregates of protein and protein-fat globules. BP and PA almond beverage presented the highest values of particle size parameters, indicating that conventional homogenization at low pressure (18 MPa) applied before heat treatment did not produce an additional decrease compared to the colloidal mill in the grinding step of almond beverage elaboration. On the other hand, PA with single effect homogenization used in this study was not enough to disperse aggregates formed into small particles.

As expected, UHPH-treated soy milk showed higher colloidal stability (Table 2) compared to pasteurized almond beverage. Particle sedimentation measured by centrifugation is indirectly related to the stability of the system. Under the same conditions, centrifugation was applied to all samples, forcing particles and aggregates to separate from the bulk, either to the top, in the case of fat globules, or to the bottom, in the case of solid particles. However, in this product with low fat concentration, measurement of the fat layer in the top was very difficult to measure in BP and PA and no creaming layer was observed at all in UHPH-treated samples. This is why the percentage of solid sedimentation was considered as indicative of the colloidal stability. This analysis can be considered as indicative of the sedimentation potential of almond beverage during long storage periods and especially as a comparative measurement among treatments applied. PA almond beverage presented a higher amount of solids settled by centrifugation than the UHPH almond beverage throughout storage (Table 2). As storage time increased, a slight increase in the percentage of sediments was observed until day 14 (maximum shelf life of PA treatment). Solid sedimentation values in UHPH samples were low and an increase was only observed at day 21 (*p* < 0.05). UHPH treatments showed lower values of this parameter; 200, 75 °C was the treatment that exhibited the best stability within the whole storage period.

**Table 2:** Solid sedimentation ^1^ of base product (BP), pasteurized (PA), and UHPH (200, 55 °C and 200, 75 °C) almond beverages at 4 °C over 21 days.

Treatment	Day 1	Day 7	Day 14	Day 21
BP	1.83 ± 0.07 ^a,A^	2.1 ± 0.05 ^a,A^		
PA	1.69 ± 0.08 ^b,B^	2.13 ± 0.62 ^a,A,B^	2.27 ± 0.14 ^a,A^	
200, 55	1.09 ± 0.11 ^c,B^	1.26 ± 0.11 ^b,B^	2.02 ± 0.28 ^a,B^	3.22 ± 0.13 ^a,A^
200, 75	0.94 ± 0.04 ^d,C^	1.01 ± 0.04 ^b,B,C^	1.15 ± 0.14 ^b,B^	3.0 ± 0.2 ^a,A^

^a−d^: Different superscripts in the same column are significantly different (*p* < 0.05); ^A−C^: Different superscripts in the same row are significantly different (*p* < 0.05); ^1^: Mean values ± SD (g/100g w/w) of solids.

### 3.3. Color Evaluation

It was observed [18] that the interaction between soy globulin fractions played an important role in the colloidal stability, mainly at high temperatures. They reported that the combination of denatured β-conglycinin and native glycinin caused lower stability of soy milk dispersion. Probably a number of different attractive particle interactions during storage are responsible for increasing solid aggregation, which was forced to sedimentation when centrifugation was applied. However, it has to be noted that those values obtained represent the maximum of destabilization. In fact, spontaneous sedimentation was negligible in UHPH samples. Solid sedimentation results indicated that the state of particle dispersion of the UHPH samples provided enough stability during 21 days of storage, revealed by solids accumulation in the bottom of the tube.

The quality of food is conditioned by many factors; some of them determine the sensory characteristics of a product. Color and appearance of foods are the first appreciations of the consumer, conditioning their preferences and influencing their choice. This characteristic is also related to chemical composition and physical changes produced during processing. Thus, color changes sometimes may determine the validity of a technology for a specific food product. Emulsion droplet characteristics have a marked influence on the appearance of this system. It was determined [19] that the lightness of an emulsion is correlated with the efficiency of the dispersion of droplets, which in turn is related to its size and its concentration of particles.

Color was measured in BP, PA, and UHPH samples of almond beverage. The effect of treatments on the CIEL*a*b* parameters, psychometric coordinates chrome (C*), and hue (h*) are shown in Table 3. Lightness (L*) is associated with the luminous intensity, which describes the light-reflecting or transmitting capacity of an object [20]. UHPH treatment caused a significant increase in the L* parameter in UHPH-treated samples compared to BP and PA almond beverages. This increase in lightness is related with the particle size reduction caused by the intensive homogenization [16], causing higher total light scatter, which has an effect on L* values’ increase. During storage, UHPH-treated almond beverage always had a higher L* value compared with PA and BP. Over time, L* values of independent almond beverage treatments remained quite stable between 1 and 21 days, especially in UHPH-treated beverages. Similar results were observed by sensory analysis, with the UHPH almond beverage being perceived as lighter than PA samples by the panel (data not shown).

**Table 3:** Effect of treatments on CIEL*a*b* parameters1 in base product (BP), pasteurized (PA), and UHPH (200, 55 °C and 200, 75 °C) almond beverages at 4 °C over 21 days.

Treatment	Day	L*	a*	b*	h*	C*	∆E_PA_
BP	1	81.4±0.2^c,B^	−0.91 ± 0.02^c,A^	7.63 ± 0.01^a,B^	83 ± 2^a,A^	7.68 ± 0.01^a,B^	6.89±0.08^a,C^
PA	1	87.58 ± 0.09^b,B^	−0.53 ± 0.02^a,A^	5.08 ± 0.07^b,A^	84 ± 2^a,A^	5.10 ± 0.07^b,A^	
200,55	1	89.3 ± 0.02^a,B^	−0.84 ± 0.02^b,A^	3.80 ± 0.01^c,A^	77 ± 3^b,B^	3.89 ± 0.01^c,A^	2.17 ± 0.05^b,B^
200,75	1	89.09 ± 0.03^a,A^	−0.96 ± 0.01^d,A^	3.47 ± 0.02^d,A^	74 ± 4^c,B^	3.60 ± 0.02^d,A^	2.25 ± 0.10^b,C^
BP	7	76.35 ± 0.01^c,D^	−1.12 ± 0.01^c,B^	6.25 ± 0.02^a,C^	79 ± 3^b,A^	6.35 ± 0.01^a,D^	8.35 ± 0.08^a,A^
PA	7	84.36 ± 0.03^b,C^	−0.72 ± 0.02^a,C^	3.93 ± 0.02^b,D^	79 ± 2^b,A^	4.00 ± 0.02^b,D^	
200,55	7	85.8 ± 0.22^a,C^	−0.96 ± 0.07^b,A,B^	2.66 ± 0.01^c,D^	67 ± 4^d,B^	2.83 ± 0.02^c,D^	1.94 ± 0.03^c,C^
200,75	7	85.71 ± 0.05^a,C^	−1.28 ± 0.01^d,C^	2.26 ± 0.02^d,D^	60 ± 4^d,B^	2.60 ± 0.02^d,D^	2.22 ± 0.02^b,C^
BP	14	79.85 ± 0.37^c,C^	−0.97 ± 0.17^a,b,A,B^	7.02 ± 0.69^a,B,C^	82 ± 2^a,A^	7.08 ± 0.70^a,C^	8.39 ± 0.34^a,A^
PA	14	87.85 ± 0.02^b,A^	−0.78 ± 0.01^a,D^	4.52 ± 0.02^b,C^	80 ± 3^a,A^	4.58 ± 0.01^b,C^	
200,55	14	89.34 ± 0.02^a,B^	−1.12 ± 0.02^b,c,B,C^	3.01 ± 0.02^c,C^	69 ± 0^d,B^	3.21 ± 0.02^c,C^	2.15 ± 0.06^b,B^
200,75	14	88.88 ± 0.02^a,B^	−1.22 ± 0.02^a,b,A,B^	2.56 ± 0.02^a,B,C^	66 ± 0^d,B^	2.83 ± 0.02^d,C^	2.26 ± 0.10^b,B^
BP	21	81.96 ± 0.16^d,A^	−0.88 ± 0.02^c,A^	8.63 ± 0.06^a,A^	84 ± 2^a,A^	8.68 ± 0.06^a,A^	7.11 ± 0.02^a,B^
PA	21	87.92 ± 0.02^c,A^	−0.65 ± 0.01^a,B^	4.77 ± 0.04^b,B^	82 ± 2^a,A^	4.82 ± 0.04^b,B^	
200,55	21	89.77 ± 0.01^a,A^	−0.83 ± 0.01^b,A^	3.24 ± 0.02^c,B^	76 ± 3^c,B^	3.35 ± 0.02^c,B^	2.40 ± 0.10^b,A^
200,75	21	89.25 ± 0.04^b,A^	−0.92 ± 0.01^d,A^	2.72 ± 0.03^d,B^	70 ± 5^d,B^	2.87 ± 0.02^c,B^	2.46 ± 0.03^b,A^

^a–c^: Different letters in the same column are significantly different (*p* < 0.05); ^A–D^: Different letters in the column for each storage period are significantly different (*p* < 0.05); ^1^: Mean values ± SD of color parameters; ΔE was calculated taking into account PA as reference sample.

The particle size distribution and concentration of UHPH and PA almond beverages were different In UHPH, a great particle size reduction (oil droplets and dispersed solid particles) was observed, with the consequent increase of interface surface produced by the increase in the number of total particles caused by treatment conditions, which in turn affected the optical characteristics of almond beverages. a* and b* color parameters in all almond beverages had negative and positive values, respectively, indicating that green and yellow were the primary contribution to the color of that beverage with predominance of the yellow component. a* values were significantly (*p* < 0.05) smaller in PA samples than UHPH samples, with (200, 75 °C) treatment being significantly higher. Over the storage period, in general, this parameter experienced an increase in their values. The contribution of b* was smaller in UHPH samples than in BP and PA samples and values of this coordinate varied during storage while maintaining the initial tendency.

Coordinate values of a* and b* were transformed in psychometric coordinates C* and h* to have a more intuitive meaning of color assessment. Those values in Table 3 indicate that BP and PA had a more marked hue of yellow and exhibited more pure color than UHPH-treated beverages. Comparing UHPH treatments, the more intense conditions caused a reduction in h* and C* values. The evolution of this color attribute during storage varied without a specific trend while maintaining the initial tendency according to treatments applied.

Color difference takes into account differences between L*, a*, and b* of the sample and a standard, in this case the PA treatment to which UHPH treatment were compared. As shown in Table 3, ΔE values were higher for BP treatments than for UHPH almond beverage. These differences may be attributed primarily to the L* parameter contribution due to treatment applied. Different results of L* and ΔE were obtained [4,5] for soy milk treated by UHPH at 200 MPa and 55 °C, 75 °C, and 40 °C of inlet temperature, respectively [21]; a significant increase in the ΔE values was reported after 21 days of storage at 4 °C of different blends of soymilk treated at 142 °C for 4 s (UHT).

### 3.4. Volatile Profile Analysis

The volatile profile is an important quality of a food product. However, treatments applied in food processing may modify volatile components to some extent, no matter whether it is beneficial or detrimental to the overall quality of food, *i.e.*, fatty acid oxidation.

Around 100 compounds were identified in almond beverages [10]. For this study, 15 of them (Table 4) were chosen, taking into consideration their abundance in the chromatographic profile and their technical interest when analyzing the influence of treatments applied (PA or UHPH) to almond beverages and the evolution of volatile profile during storage. Among them, the most representative compounds in almond beverage head space were aldehydes (benzaldehyde, hexanal, and nonanal), alcohols (1-pentanol, 1-hexanol, 1-heptanol, 1-nonanol, 1-octen-3-ol, 1-nonanol, 2-etil-1-hexanol, benzeneethanol, and benzyl alcohol), and some from different families (toluene, styrene, and benzoic acid).

**Table 4:** Abundance^1^ of selected volatile compounds in base product (BP), pasteurized (PA), and UHPH (200, 55 °C and 200, 75 °C) almond beverages at 4 °C over 21 days.

Compound	Day 1	Day 7	Day 14	Day 21
***Benzaldehyde***				
PA	283.45 ^A,a^	194.22 ^A,b^	181.60 ^B,b^	
200, 55	304.28 ^A,a^	289.02 ^A,a^	206.20 ^A,a^	177.85 ^A,a^
200, 75	283.51 ^A,a^	197.09 ^A,a^	202.18 ^A,a^	175.45 ^A,a^
***Hexanal***				
PA	53.08 ^A,a^	20.71 ^B,b^	18.60 ^B,b^	
200, 55	100.33 ^A,a^	67.27 ^A,a^	68.92 ^A,a^	41.68 ^A,a^
200, 75	114.80 ^A,a^	94.52 ^A,a^	96.69 ^A,a^	52.32 ^A,a^
***Nonanal***				
PA	3.37 ^A,a^	2.85 ^A,b^	2.23 ^B,b^	
200, 55	4.12 ^A,a^	3.51 ^A,a^	4.74 ^A,a^	4.10 ^A,a^
200, 75	5.58 ^A,a^	4.99 ^A,a^	6.13 ^A,a^	8.81 ^A,a^
***1-pentanol***				
PA	23.03 ^B,a^	22.78 ^A,a^	19.77 ^B,a^	
200, 55	29.26 ^A,a^	24.16 ^A,a^	27.98 ^A,a^	20.16 ^A,a^
200, 75	31.38 ^A,a^	31.45 ^A,a^	28.89 ^A,a^	24.30 ^A,a^
***1-hexanol***				
PA	60.66 ^A,b^	99.28	96.18 ^A,a^	
200, 55	42.89 ^B,b^	39.50 ^B,b^	114.4 ^A,a^	93.45 ^A,a^
200, 75	36.77 ^B,a^	69.45 ^A,B,a^	67.60 ^A,a^	65.26 ^A,a^
***1-heptanol***				
PA	11.74 ^A,b^	11.19 ^A,a,b^	8.71 ^A,b^	
200, 55	18.88 ^A,a^	15.43 ^A,a^	20.09 ^A,a^	16.63 ^A,a^
200, 75	26.73 ^A,a^	20.75 ^A,a^	18.64 ^A,a^	26.73 ^A,a^
***1-nonanol***				
PA	3.27 ^A,a^	3.03 ^A,a^	2.16 ^A,b^	
200, 55	1.66 ^B,a^	1.70 ^B,a^	2.62 ^A,a^	1.92 ^A,a^
200, 75	1.77 ^B,a^	1.71 ^B,a^	1.35 ^B,a^	1.68 ^A,a^
***1-octen-3-ol***				
PA	19.51 ^B,ab^	19.60 ^B,a^	14.39 ^A,b^	
200, 55	22.72 ^A,a^	21.14 ^B,a^	26.99 ^A,a^	21.38 ^A,a^
200, 75	38.95 ^A,B,a^	34.53 ^A,a^	40.47 ^B,a^	38.97 ^A,a^
***2-etil-1-hexanol***				
PA	5.15 ^A,b^	9.24^A,a^	5.56 ^A,b^	
200, 55	5.55 ^A,a^	4.12^B,a^	5.37 ^A,a^	3.99 ^A,a^
200, 75	3.89 ^B,a^	5.29^A,B,a^	5.08 ^A,a^	4.46 ^A,a^
***Benzeneetanol***				
PA	40.31 ^A,b^	30.54 ^A,c^	65.20 ^A,a^	
200, 55	35.11 ^A,B,a^	33.48 ^A,a^	62.63 ^A,a^	60.29 ^A,a^
200, 75	30.33 ^B,a^	29.85 ^A,a^	26.94 ^B,a^	28.61 ^A,a^
***Isoamyl alcohol***				
PA	3.29 ^A,a,b^	3.88 ^A,a^	2.54 ^A,b^	
200, 55	2.18 ^B,b^	1.88 ^B,b^	3.22 ^A,a^	2.75 ^A,a^
200, 75	1.70 ^B,a^	1.96 ^B,a^	1.79 ^A,a^	1.71 ^A,a^
***Benzyl alcohol***				
PA	4.50 ^A,b^	28.55 ^A,a^	28.88 ^A,a^	
200, 55	8.62 ^A,b^	12.67 ^Ab^	66.44 ^A,a^	55.62 ^A,a^
200, 75	8.01 ^A,a^	49.66 ^A,a^	49.54 ^A,a^	49.70 ^A,a^
***Tolueno***				
PA	6.85 ^A,a,b^	8.91 ^A,b^	5.97 ^A,a^	
200, 55	3.85 ^B,a^	4.06 ^A,a^	4.48 ^A,a^	3.75 ^A,a^
200, 75	4.04 ^A,a^	6.34 ^A,a^	3.94 ^A,a^	3.74 ^A,a^
***Estyrene***				
PA	0.67 ^A,a^	0.70 ^A,a^	0.59 ^A,b^	
200, 55	1.60 ^B,a^	1.41 ^A,a^	1.44 ^A,a^	1.48 ^A,a^
200, 75	1.50 ^A,B,a^	1.21 ^A,a^	1.13 ^A,a^	1.45 ± 1.06 ^A,a^
***Benzoic acid***				
PA	3.57 ^A,b^	1.84 ^B,b^	1.69 ^A,a^	
200, 55	3.37 ^A,B,a^	3.15 ^A,a^	1.65 ^A,a^	1.62 ^A,a^
200, 75	2.60 ^B,a^	1.85^A,B,a^	1.79 ^A,a^	1.59 ^A,a^

^a–b^: Different letters in the same row are significantly different (*p* < 0.05); ^A–C^: Different letters in the same column for a same compound are significantly different (*p* < 0.05); ^1^: Integrated area counts. Mean value × 10^5^.

In general, UHPH conditions applied to almond beverages did not show significant differences in the volatile profile; it could be considered that both UHPH treatments (200, 55 °C and 200, 75 °C) were equivalent from this point of view. However, PA-treated samples exhibited differences in most compounds, making them different (*p* < 0.05) to UHPH-treated almond beverages. The effect of UHPH and PA treatments on volatile compounds evolution during storage is shown in Table 4. Benzaldehyde was the most abundant in the chromatographic profile of all samples, as expected since it is a “character-impact compound” in almonds, providing a pleasant almond-like flavor. Benzaldehyde can be produced naturally from the action of β-glucosidases, and can be thermally generated from phenylalanine [22]. Upon oxidation, benzaldehyde is transformed into benzoic acid and, via hydrogenation, into benzyl alcohol. This is coherent with the evolution of those compounds during storage, especially with benzyl alcohol evolution from day 1 on.

The generation of certain volatiles, such as hexanal and nonanal, may be used to monitor the oxidation of a food product. Hexanal is a good indicator of lipid oxidation for foods rich in omega-6 fatty acids [23]. The fatty acid composition of almonds is rich in linoleic acid, an omega-6 fatty acid. Analysis of hexanal pick area integration on day 1, as a first approximation, indicates that UHPH treatment cause higher oxidation than pasteurization, although statistical analysis show no differences (*p* > 0.05) among treatments, which can be attributed to the high variability found in the analysis of this compound, probably due to the effect of the different almond beverage productions. Taking into account the general production process of almond beverages, the most probable factors affecting lipid oxidation are high temperatures and the lipid exposition to catalyst agents. In UHPH treatments, and especially in the conditions applied in this study, the maximum temperature reached was 115 °C during a very short time (less than one second); thus the most probable negative effect on lipid oxidation was the high degree of exposure of the lipid fraction due to the fat droplet reduction and the resulting increase in surface area.

Over the storage period, a significant decrease (*p* < 0.05) was observed in hexanal content in all studied samples. Hexanal may be degraded to carboxylic acid in presence of oxygen [24] and may also be transformed in 1-hexanol by reduction [25], which is in line with the increase of this compound over storage.

Bezene etanol, 2-etil-1-hexanol, and benzyl alcohol also experienced a significant increase over the storage period (*p* < 0.05). These compounds probably originated as secondary products from lipid oxidation, as mentioned by the authors of [26], who found them in roasted almonds. The fat fraction in almonds is about 40% and the fatty acid profile is rich in mono- and polyunsaturated fatty acids. To produce almond beverage, the fat fraction has to be liberated by heat wet milling to obtain the coarse emulsion prior to homogenization and heat treatment for preservation of the final product. Thus, lipid degradation compounds contribute to the characteristic volatile profile of processed almonds.

## 4. Conclusions

UHPH is a potential alternative to conventional pasteurization, producing high stable vegetable beverages from a microbiological and physical point of view. Chemically, UHPH treatment negatively affected lipid oxidation, which has to be more extensively studied to evaluate the possibility of adding antioxidant agents and their impact on this quality parameter. In terms of microbiological quality, the mildest conditions of UHPH applied (200 MPa with 55 °C inlet temperature) were similar to pasteurization and 200 MPa, 75 °C considerably improved the hygienic characteristics of almond beverages, producing the best microbial stability during cold storage. At least one week of extended shelf life was obtained for almond beverage compared to that of pasteurized (90 °C, 90 s), with good overall quality.
